# Fallopian Tube Torsion as a Cause of Acute Pelvic Pain in Adolescent Females

**DOI:** 10.1155/2016/8707386

**Published:** 2016-10-13

**Authors:** Claudia Mueller, Sandra Tomita

**Affiliations:** ^1^Department of Surgery, Stanford University School of Medicine, Stanford, CA, USA; ^2^Department of Surgery, New York University School of Medicine, New York, NY, USA

## Abstract

*Purpose*. Torsion of the fallopian tube, involving hydatids of Morgagni, though a rare cause of acute pelvic pain in young girls, can pose significant risks to future fertility. Tubal torsion may present as a diagnostic dilemma since the ovary itself usually appears normal on ultrasound. Thus, surgical intervention may be delayed which can lead to worsening necrosis and result in the need for resection of the affected tube.* Methods*. We reviewed two cases of fallopian tube torsion associated with hydatids of Morgagni in adolescent females.* Results*. The patients were premenarchal in both cases, aged 10 and 13 years. Both presented with acute clinical signs of ovarian torsion but ultrasound showed the ovary itself to be normal with an adjacent cystic structure. In both cases, the fallopian tube was detorsioned laparoscopically and preserved. The associated cyst was excised in one case and marsupialized in the other.* Conclusions*. We propose that prompt recognition and operative management of this relatively uncommon source of pelvic pain may prevent unnecessary tubal resection and improve long-term fertility in this population.

## 1. Introduction

Hydatids of Morgagni (HMs) are pedunculated cystic structures which arise from the fimbriated ends of the fallopian tubes [1]. These Müllerian duct remnants are usually filled with serous fluid and most measure less than two centimeters [1]. Typically benign, HMs are often noted incidentally during pelvic surgery, but their presence can become of some clinical concern. For instance, HMs, especially when they occur bilaterally, have been implicated as a factor in infertility [2]. Furthermore, the existence of HM at the end of a fallopian tube may increase the likelihood of tubal torsion, particularly in adolescent females [3].

Fallopian tube torsion, while far less common than ovarian torsion, may present in a remarkably similar fashion. Isolated fallopian tube torsion has an incidence reported to be 1 in 1.5 million in adult women [4]; however, its occurrence in the pediatric or adolescent population is even less certain with approximately 35 cases described in the literature. Tubal torsion has been linked to hydrosalpinx as well as paratubal cysts, including HMs [5–8].

The presentation of fallopian tube torsion is similar to that of ovarian torsion: abdominal or pelvic pain which may be associated with fever, nausea, or vomiting [3, 5, 8–12]. The pain may be acute or chronic and is sometimes less well localized than that of ovarian torsion [8]. Laboratory values are often within normal limits. Fallopian tube torsion can be difficult to demonstrate radiographically as the ovary usually appears normal on ultrasound and a twisted tube may be easily missed [8]. HMs are even more difficult to identify radiologically, given their usual small size, and may often be misclassified as simple ovarian cysts [13].

Given the sometimes vague nature of the presenting symptoms and often poor imaging diagnostics, surgical intervention for fallopian tube torsion can be delayed, which may lead to more advanced necrosis with the need for resection of the affected tube. Laparoscopy appears to provide an excellent mechanism for early diagnosis and treatment of fallopian tube torsion. We describe two cases of fallopian tube torsion, both associated with HM, treated laparoscopically in adolescent females.

## 2. Methods/Patients

Our two patients, aged 10 and 13 years, were both premenarchal. They each presented to the Emergency Department with acute pelvic pain of several hours' duration. The pain was colicky and localized to the pelvis with no previous episodes. Neither described gastrointestinal or urinary symptoms. There was no history of trauma or pertinent previous medical or family history. Neither patient was sexually active.

Both patients were afebrile with stable vital signs. Physical exams were normal except for lower abdominal tenderness, on the right in one patient and on the left in the other. There were no palpable masses. Laboratory tests for both patients, including urinalysis, were normal. Ultrasonography was performed transabdominally on both patients, given their young age. Both showed normal ovaries bilaterally, with appropriate blood flow. Adnexal cysts were described in both cases, on the left in one patient and on the right in the other.

Intravenous hydration and pain medication were given to both patients, but both had persistent pain and so were taken to the operating room for diagnostic laparoscopy within that day.

**Figure 1:**
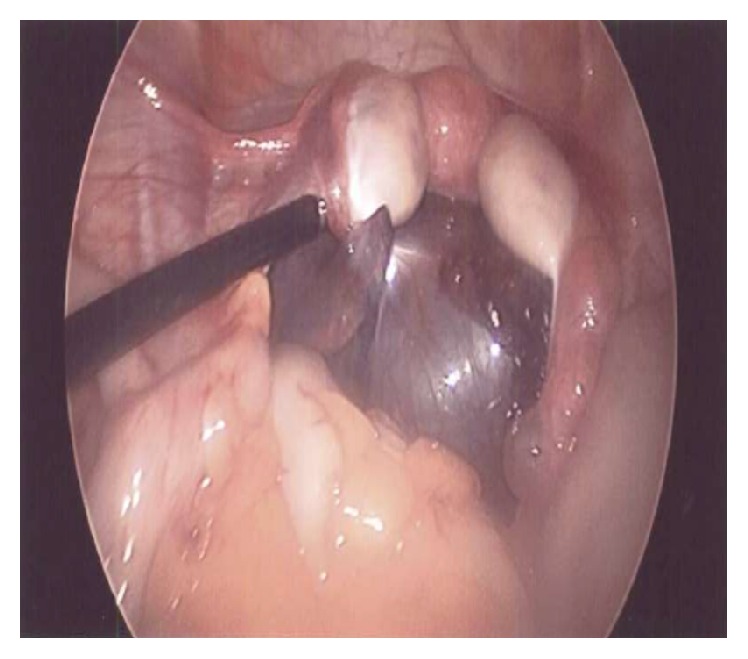
Laparoscopic view of torsed left fallopian tube.

**Figure 2:**
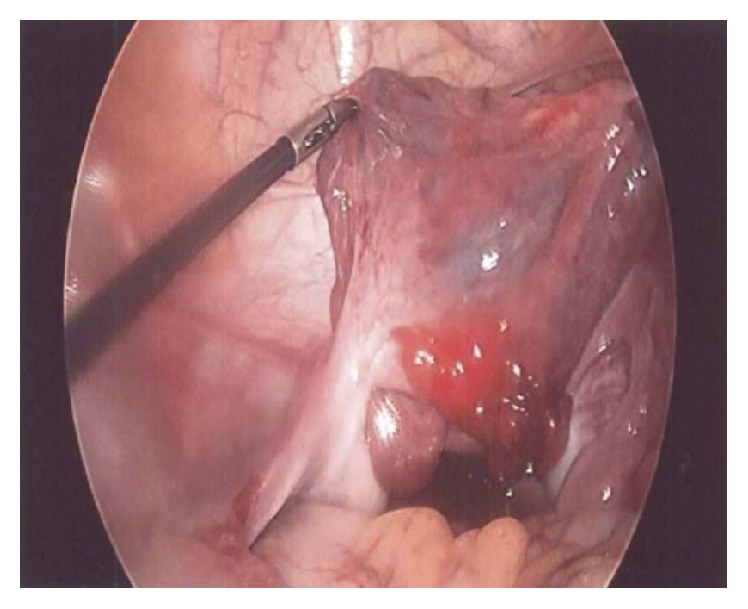
Laparoscopic view of detorsioned left fallopian tube with paratubal hydatid of Morgagni.

Isolated torsion of the fallopian tube with ischemia was noted on both patients (Figure 1). The ovaries were not involved in the torsion and appeared normal. Both tubes were able to be detorsioned laparoscopically with return of blood flow (Figure 2). Upon detorsion, one HM was identified at the fimbriated end of the torsed tube in each patient. No bilateral HMs were noted.

After the fallopian tubes were detorsioned, the HMs were addressed. The cyst was excised in one patient and marsupialized in the other. No necrosis of the torsed tubes was noted and so both were left in place.

Both patients had an unremarkable recovery and were discharged by postoperative day two.

## 3. Discussion

Fallopian tube torsion, while much less common than ovarian torsion, has been described previously as a cause of pelvic pain in adolescent females. Causes of this disease entity are unknown but are likely associated with alterations in tube balance or rotation which can be associated either with hydrosalpinx or, even more rarely, with paratubal cysts such as HMs [3]. Mechanisms for fallopian tube torsion caused by HM have been linked to entanglement of HM's pedicle with the fallopian tube or increases in heaviness of end of the tube caused by the HM's location at the fimbria [3]. There has been a suggestion that HMs may increase in size because of secretory activity in puberty, which may lead to increases in the likelihood of tube rotation and torsion [3].

Whatever the origin, because of its relative rarity, fallopian tube torsion is often not considered as a potential cause of abdominal pain in adolescent females. The diagnostic difficulty can be compounded because the most commonly used imaging study in this young, usually sexually inactive, population is transabdominal ultrasonography which may show normal ovaries, leading clinicians to abandon a diagnosis of adnexal torsion. Other imaging modalities such as CT scan or MRI have disadvantages such as radiation exposure, cost, or lack of ready availability which render them potentially less useful for rapid diagnosis. Delays in diagnosis may increase the likelihood of necrosis of the fallopian tube which would result in salpingectomy as has been described in earlier reports of isolated tube torsion [5].

Thus, we propose that the consideration of laparoscopy as a diagnostic tool for acute onset pelvic pain in the absence of ovarian pathology may be especially relevant in adolescent females. Prompt recognition and intervention may allow for laparoscopic detorsion and cyst excision, rather than tube resection, which would help preserve fertility in this population.
